# An Optimized Method for Manufacturing a Clinical Scale Dendritic Cell-Based Vaccine for the Treatment of Glioblastoma

**DOI:** 10.1371/journal.pone.0052301

**Published:** 2012-12-20

**Authors:** Sara Nava, Marta Dossena, Simona Pogliani, Serena Pellegatta, Carlo Antozzi, Fulvio Baggi, Cinzia Gellera, Bianca Pollo, Eugenio A. Parati, Gaetano Finocchiaro, Simona Frigerio

**Affiliations:** 1 Cell Therapy Production Unit – UPTC, Fondazione IRCCS Istituto Neurologico Carlo Besta, Milan, Italy; 2 Unit of Molecular Neuro-Oncology, Fondazione IRCCS Istituto Neurologico Carlo Besta, Milan, Italy; 3 Neurology IV, Fondazione IRCCS Istituto Neurologico Carlo Besta, Milan, Italy; 4 Department of Genetics of Neurodegenerative and Metabolic Diseases, Fondazione IRCCS Istituto Neurologico Carlo Besta, Milan, Italy; 5 Neuropathology Unit, Fondazione IRCCS Istituto Neurologico Carlo Besta, Milan, Italy; Tulane University, United States of America

## Abstract

Immune-based treatments represent a promising new class of therapy designed to boost the immune system to specifically eradicate malignant cells. Immunotherapy may generate specific anti-tumor immune responses, and dendritic cells (DC), professional antigen-presenting cells, are widely used in experimental cancer immunotherapy. Several reports describe methods for the generation of mature, antigen-pulsed DC for clinical use. Improved quality and standardization are desirable to obtain GMP-compliant protocols. In this study we describe the generation of DC from 31 Glioblastoma (GB) patients starting from their monocytes isolated by immunomagnetic CD14 selection using the CliniMACS® device. Upon differentiation of CD14+ with IL-4 and GM-CSF, DC were induced to maturation with TNF-α, PGE_2_, IL-1β, and IL-6. Whole tumor lysate was obtained, for the first time, in a closed system using the semi-automated dissociator GentleMACS®. The yield of proteins improved by 130% compared to the manual dissociation method. Interestingly the Mean Fluorescence Intensity for CD83 increased significantly in DC pulsed with “new method” lysate compared to DC pulsed with “classical method” lysate. Our results indicate that immunomagnetic isolation of CD14^+^ monocytes using the CliniMACS® device and their pulsing with whole tumor lysate proteins is a suitable method for clinical-scale generation of high quality, functional DC under GMP-grade conditions.

## Introduction

Glioblastoma (GB) is one of the most aggressive forms of cancer and the most common primary malignancy in the central nervous system. Current treatment remains palliative [1], [2], therefore novel therapies are greatly needed.

Numerous animal models indicate that Dendritic Cells (DC) are effective in the induction of therapeutic antitumor responses [3], [4], [5] and clinical trials indicate their efficacy in human pathologies [6], [7] showing that effective immune responses within the CNS can be generated through the use of DC-based vaccines [8].

Immunotherapy with DC incubated with tumor lysate or peptides seems capable of generating a specific anti-tumor immune response [9], [10], it is biologically safe without serious side effects noted in pre-clinical or clinical trials [11], [12]. The development of methods to generate DC in accordance with good manufacturing practice (GMP) guidelines is mandatory [7]. Two phase-I clinical studies sponsored by the Istituto Neurologico Carlo Besta (DENDR1, Eudract 2008-005035-15; DENDR2, Eudract 2008-005038-62) for DC-based immunotherapy of GB have been approved by the Italian Ministry of Health.

Since circulating DC are few, representing only 0.1–1% of peripheral blood mononuclear cells (PBMC), large amounts of DC must be obtained *in vitro* from CD14+ monocytes purified from leukapheresis or buffycoat [13].

Differentiation of CD14+ into DC can be obtained in 7 days by utilizing GM-CSF and IL-4 [14]; DC can be induced to maturation in 24 h with a cocktail of pro-inflammatory cytokines [15].

Immune response specificity induced by DC is based on pulsing with tumor lysate that contains a multiple and unaltered spectrum of known and unknown tumor antigens which are patient-specific [16]. To ensure reproducible results in lysate preparation, we modified the tissue homogenization procedure for protein extraction using GentleMacs Dissociator, a closed system providing an increase in the yield of protein extraction.

The whole process is performed in the Clean-room facility of the “Cell Therapy Production Unit” in the Istituto Neurologico Carlo Besta.

This method is reproducible and conforms to GMP guidelines for pharmaceutical products, as assessed by microbiological safety, viability, phenotype and functionality of DC produced.

## Materials and Methods

### DC Culture

The study was approved by the local institutional review board of the Fondazione IRCCS Istituto Neurologico Carlo Besta (Milan, Italy) and informed written consent was obtained from all patients.

Patients underwent leukapheresis procedure without prior cytokine stimulation, using a closed system (AutoPBSC, Specra Cell Separator, CaridianBTC).

The leukapheresis product was washed with PBS/EDTA (Miltenyi) and centrifuged at 200×g for 10 minutes. Total whole blood cells (WBC) number and monocytes percentage in the leukapheresis were evaluated by hemogram analysis. WBC were incubated with anti-CD14-conjugated beads (Miltenyi) for 15 minutes at room temperature, washed with PBS/EDTA (Miltenyi) and centrifuged at 200×g for 10 minutes. CD14+ cells were sorted on CliniMACS® system (Miltenyi). Positive fraction was cultured at 3–5×10^6^ cells/ml in VueLife® closed culture systems (Afc) in CellGRO Medium (CellGenix), implemented with 20 ng/ml IL-4 and 50 ng/ml GM-CSF (CellGenix).

All reagents were for clinical use only.

On day 5 of culture, immature DC (iDC) were pulsed with autologous tumor lysate at the concentration of 50 µg/10^6^ living cells plus 50 µg/ml keyhole limpet hemocyanin (KLH, Calbiochem) with addition of 10 ng/ml IL-4 and 25 ng/ml GM-CSF for 24 hours.

On day 6, antigen-loaded DC (aDC) were cultured with pro-inflammatory cytokine cocktail including: 10 ng/ml of TNF-α, IL-1β, IL-6 (CellGenix) and 1 µg/ml PGE_2_ (Pfizer).

After 24 h, mature antigen-loaded DC (mDC, final product), were collected and frozen at the concentration of 5–6×10^6^ viable cells/vial in NaCl (B.Braun), 10% dimethyl sulfoxide (DMSO, Listarfish), and 5% human albumin (Kedrion). A controlled-rate freezer curve (Planer Kryo 360-3.3, Planer Products) was used prior to preservation in nitrogen gas until use.

All samples were stored in the GMP dedicated area of the bio-bank and managed with a Good Automated Manufacturing Practices-4 (GAMP 4) software.

The schematic representation of the process is summarized as Flow Chart in Figure S1.

### Tumor Protein Lysate

GB specimens removed during surgery were divided in two representative portions. One portion was devoted to histopathological analysis, the other was used for the production of protein lysate. Tissue requirements were as follow: minimum weight 1 g; tissue necrosis less than 20%, no presence of non-tumor tissue. Samples dedicated to production of protein lysate were washed in sterile NaCl 0.9% solution, weighed and snap-frozen in nitrogen gas until use.

For tumor lysate preparation two alternative methods were adopted.

#### “Classical” method

Tumor protein extraction was performed as previously described by Ashley and colleagues [17].

Tumor samples were minced and mechanically dispersed using decreasing size needles (18G, 20G and 21G). Single cell suspension was diluted in PBS (Listarfish) and centrifuged at 300×g for 5 minutes. Pellet was resuspended in PBS and percolated through a 70 µm and 30 µm filter. The suspension was centrifuged at 300×g for 5 minutes and sonicated in a bath for at least 1 hour (Elmasonic S10, Elma).

#### New method

Tumor samples were minced, transferred to Tube type C (Miltenyi) and mechanically dispersed with GentleMACS™ Dissociator (Miltenyi) using installed software program “m_spleen04”. Single cell suspension was centrifuged at 1000×g for 10 minutes. Pellet was re-suspended in PBS, relocated in a Tube type M (Miltenyi) and lysed through GentleMACS™ Dissociator using program “protein_01″. Suspension was percolate through 70 µm and 30 µm filter ant then sonicated for 30 minutes.

For both methods: the presence of latent live tumor cells was determined by trypan blue exclusion. If viable cells were present, further sonication steps were performed until 0% viability was obtained. Protein content was determined by reaction with bicinchoninic acid (BCA) (Pierce Biotechnology/Thermo Fisher Scientific) following manufacturer instructions.

The schematic representation of the two processes is summarized as Flow Chart in Figure S2.

### Flow Cytometry

FACS analysis was performed for CD14, CD80, CD83, CD86 and HLA-DR molecules on PBMC from healthy donors and DC harvested at different culture steps (iDC day 5, aDC day 6, mDC day 7). 3×10^5^ cells/tube were stained with fluorochrome conjugated mAbs (BD) and incubated for 30 minutes at 4°C in the dark. Aspecific staining was determined with appropriate Isotype Control (BD).

Samples were centrifuged at 300×g for 5 minutes, washed twice with cold FACS buffer and analyzed immediately, or after fixation with 4% paraformaldehyde, in a FACSCalibur flow cytometer (BD) equipped with CellQuest software. At least 20,000 events were acquired for each sample. Non-viable cells were excluded by physical gating.

### Mixed Lymphocyte Reaction (MLR)

PBMC were isolated from patient or unrelated healthy donor blood by centrifugation over a Ficoll-Paque™ gradient and re-suspended in CellGRO medium (CellGenix).

Unidirectional MLRs were performed by co-culturing 2×10^5^ PBMC (responder cells: R) with stimulating cells (S) in a 96-well plate (Corning). “S” cells were represented by: 1×10^4^ DC, 2×10^5^ autologous PBMC (for auto-MLR, negative control) or 2×10^5^ allogeneic PBMC (for allo-MLR, positive control).

“S” cells were pre-treated with mitomycin-C (50 µg/ml, Sigma Aldrich) for 20 minutes at 37°C and used after extensive wash.

After 5 days, 1 µCi [^3^H]-thymidine (Amersham Biosciences) was added for further 18 h. The radioactivity incorporated into DNA was measured in a β-scintillation counter (Trilux 1450, Wallac/Perkinelmer). Results were expressed as stimulation index (SI) to allow comparison of results between donors. SI was calculated as follow: mean counts per minute (cpm) from stimulated cells/mean cpm from non-stimulated cells. MLR responses were considered positive when SI≥3 for PBMC-induced stimulation and SI≥6 for DC-induced stimulation.

### Statistical Analysis

The results were expressed as mean±SD. One way anova and two tailed test was utilized for all statistical analyses and performed with GraphPad Prism software, version 4.0. P values of less than 0.05 were considered to be significant.

## Results

### Purification of Monocytes, DC Yield and Viability

To assess the purity of CD14+ fraction, selected monocytes were analyzed by flow cytometry for CD14 expression. Analysis of 31 separations demonstrated a mean purity of 98.7±2.3% (SD) after immuno-selection.

The present protocol was designed to prepare DC vaccines for GB patients providing a total amount of 55×10^6^ mDC for repeated administrations. At least 5×10^9^ of starting WBC were necessary to obtain 55×10^6^ DC at the end of the procedure. During the past two years 13 first diagnosis GB patients (DENDR1) and 18 recurrent GB patients (DENDR2) have been enrolled in the clinical trial. Considering 31 events of leukapheresis, an average±SD of (11.2±4.0)×10^9^ WBC with a mean monocyte percentage of 13.0±5.4% was obtained. mDC obtained at the end of the procedure were (117.1±347.0)×10^6^, representing a yield of 1.2±0.7% on the total WBC or 9.1±3.1% on the cultured CD14+.

The amount of tumor lysate is critical for the identification of the final number of iDC to be carried on for subsequent culture passages (total iDC or fraction). When the tumor lysate was sufficient to load only a fraction of iDC, cells in excess were cultured in parallel without antigen. Tumor-unloaded iDC were also considered for the calculation of the final yield of each production.

No statistically significant differences were observed in terms of yield and viability between DENDR1 and DENDR2 preparations. Data are summarised in Table 1.

**Table 1:** Summary of DC production data.

					mDC Yield		
Protocol	# of productions	(a)WBC ×10^9^	(b)cultured CD14^+^×10^9^	(c)mDC ×10^6^	(d)% on WBC	(e)% on CD14^+^	
**All**	31	11.52±4.0	1.3±0.5	117.1±47.0	1.2±0.7	9.1±3.1	
**DENDR1**	13	12.61±3.7	1.4±0.5	122.2±36.3	1.1±0.4	9.5±2.7	(f) **p = **
**DENDR2**	18	10.7±4.6	1.23±0.7	114.1±49.2	1.2±0.7	8.8±2.7	**ns**

(a) number of WBC present in the starting leucapheresis.

(b) number of CD14^+^ cells obtained after CliniMACs selection.

(c) number of mDC obtained at the end of culture.

(d) yield of mDC respect to the starting WBC.

(e) yield of mDC respect to the cultured CD14^+^.

(f) ns = Not significant (p>0.05) for all the parameter evaluated.

mDC were maintained until use in nitrogen gas, after controlled freezing ramp; the controlled reduction of temperature improved vitality after cell thawing, ensuring a minimum of 75% live cells. Every batch product resulted in a final viability higher than 85% (range 86%–96%, mean 94.3±3.9%). A representative example of the controlled-rate freezer curve is shown in supplementary data (Figure S3).

### Preparation of Tumor Protein Lysate

The improvement in lysate preparation guarantees an amount of tumor proteins sufficient to load all iDC in culture in all DC preparations.

**Figure 1:**
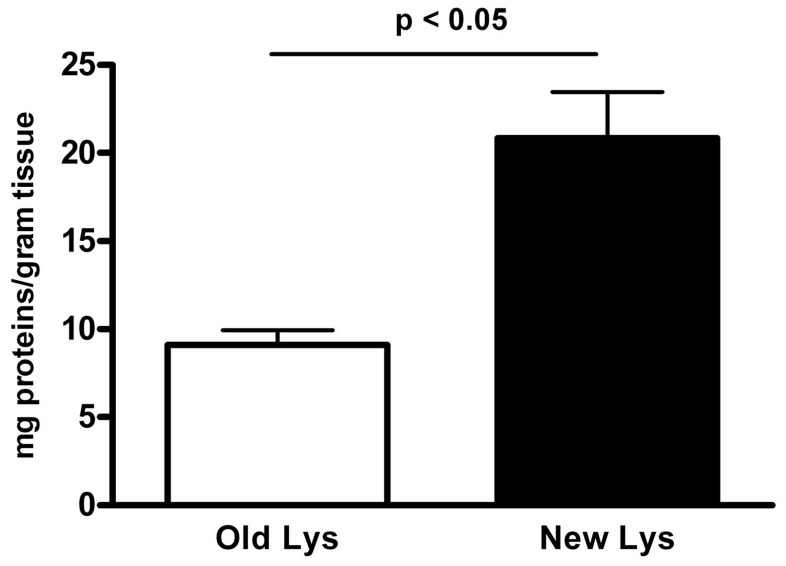
Lysate protocol. The modified lysate protocol (New method, black bar, 19 productions) increased the yield of protein obtained per gram of tissue by 130% with respect to the Old “Classical method” (empty bar, 12 productions). Data are represented as mean mg of proteins per gram of tissue. Statistical bars on graph indicate the SD value.

As shown in Fig. 1, the optimized lysate protocol increased protein yield obtained per gram of tissue by 130% with respect to the “classical method” [17]. Analysis of Protein content indicated a mean of 20.9±2.6 mg of protein per gram of tissue for the “new method” (19 preparations) compared to 9.1±0.8 for the “classical method” (12 preparations, p<0.05).

### Phenotypic Evaluation of iDC, aDC and mDC

**Figure 2:**
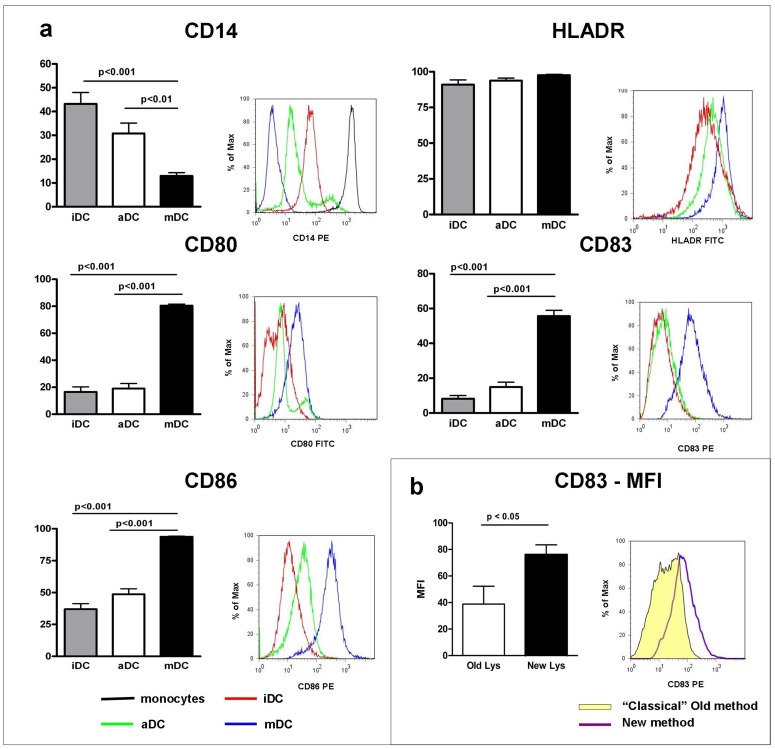
Phenotypic analysis of DC. a) Phenotypic analysis of maturation-associated markers was determined by flow cytometry on iDC, aDC and on mDC. Variation of the percentage of positive cells expressing maturation markers (CD80, CD83, CD86) and down-regulation of CD14 (monocytes marker) are represented. A statistically significant difference for maturation markers was present between mDC (black bar) and both iDC (gray bar) and aDC (empty bar) (p<0.05); no differences were observed in HLA-DR expression. Statistical bars on graph indicate the SD value. The overlay representation of histograms illustrates the up regulation of CD80, CD83, CD86 and the down regulation of CD14 during culture. Only a slight up-regulation was observed on HLA-DR expression during maturation stages (p>0.05). Histograms are representative of one of 31 independent productions analysed. iDC (red line); aDC (green line); mDC (blue line); monocytes (black line). **b)** CD83 expression resulted higher and more homogeneous in DC activated with tumor lysate produced following the “new method” (black bar, 19 productions) than in DC activated with tumor lysate produced following the old “classical method” (empty bar, 12 productions). Data are expressed as MFI normalized on isotype control. The overlay representation of histograms reveals that CD83 expression resulted more homogeneous in the second group of DC (“new method”, purple line) respect to the first one (old “classical method”, yellow filled).

The expression of surface markers during different maturation stages of DC was assessed by flow cytometry. Expression profiles were evaluated on iDC, aDC and mDC, as represented in Fig. 2a.

The analysis included the up-regulation of the typical maturation markers CD80, CD83 and CD86 and the down-regulation of CD14, the marker of undifferentiated precursor cells; HLA-DR was considered as a control marker for antigen presenting cells. Maturation analysis demonstrated that CD80, CD83 and CD86 expression increased significantly: CD83 (8.15±10.6 iDC vs 55.6±19.1 mDC;p<0.001), CD80 (16.5±21.6 iDC vs 80.4±6.4 mDC; p<0.001) and CD86 (37.0±23.0 iDC vs 93.8±2.4 mDC; p<0.001).

Conversely, CD14 expression decreased from 43.2±26.3% in iDC to 12.9±7.6% in mDC (p<0.001).

HLA-DR expression maintained a constant level during culture, presenting a slight up-regulation between iDC and mDC (91.0±14.7% and 97.6±2.7%, ns).

No statistically significant differences for analyzed markers were observed between iDC and aDC.

### The Production of Tumor Lysate following the “New Method” Improved the Final Maturation of DC

The phenotypical analysis of DC pulsed with the “new method-tumor lysate” demonstrated that the expression of CD83 molecule is higher and more homogeneous than in DC activated with the “classical method-tumor lysate” (Fig. 2b). Interestingly, FACS analysis showed that “new method” DC expressed CD83 with an average Mean Fluorescence Intensity (MFI) of 75.3±8.1, while “classical method” DC expressed CD83 with an average MFI of 38.9±13.4 (p<0.05). Data were normalized on MFI of isotype control.

No statistically significant differences for other phenotype markers analyzed were observed between mDC activated with “new method-tumor lysate” or “classical method-tumor lysate”.

### Evaluation of the Functionality of iDC and mDC

**Figure 3:**
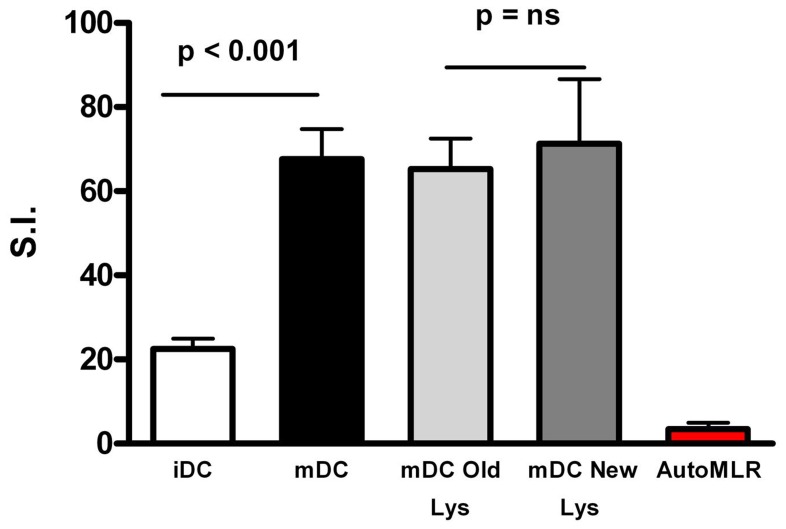
Functional evaluation of DC. The ability of iDC versus mDC in the antigen presentation was compared evaluating the potency of DC as their *in vitro* allo-stimulatory capacity of PBMC from healthy volunteers. Final product (mDC, black bar, 31 experiments) resulted more potent than its immature counterpart (iDC, empty bar, 18 experiments) in the MLR induction. We compared new lysate activated and old lysate activated cells for their ability in the induction of allogeneic MLR functional assay. “New method-tumor lysate” (19 productions) activation seem to slightly improve the functional ability of mDC in their allo-stimulatory ability respect to “classical method-tumor lysate” (12 productions) activation but this difference is not statistically significant. By contrast no proliferative responses were induced by antigen-loaded mDC in the lymphocyte of GB patients prior to vaccination (red bar, 18 experiments). Data are expressed as Stimulation Index (SI); statistical bars on graph indicate the SD value.

DC functionality was evaluated via one-way MLR using PBMC as responder cells in iDC and mDC. As shown in Fig. 3, mDC were more potent than their immature counterpart in allo-stimulatory capacity, with a mean SI±SD of 67.6±7.2 in mDC and 22.5±2.4 in iDC, (p<0.001).

We compared new lysate activated and old lysate activated cells for their ability in induction of allogeneic MLR functional assay. As Shown in Fig. 3 “new method-tumor lysate” activation seem to slightly improve the functional ability of mDC in their allo-stimulatory ability respect to “classical method-tumor lysate” activation (mean SI±SD of 65.3±7.3 in mDC “old lysate” and 71.3±15.3 in mDC “new lysate”) but this difference is not statistically significant.

Moreover, we evaluated the proliferation induced by mDC in autologous PBMC from patients before vaccination. Data shown in Fig. 3 indicate that no proliferation is induced by antigen-loaded mDC in the lymphocytes of GB patients prior to vaccination. These data are preliminary to the immuno-follow-up of the vaccinated patients (Pellegatta et al, manuscript under review).

## Discussion

In the last decade a growing attention has been placed on the generation of DC suitable for clinical applications. A number of papers reported protocols for reproducible monocyte-derived DC generation [14], [15]; particularly in 2003 Babatz et al described a GMP-like approach based on the Large-Scale Immunomagnetic Selection of CD14+ Monocytes [18]. In our study we introduced three innovative changes in methods for DC preparation: i) processing of the tumor performed with an automated, closed system; ii) DC differentiation, loading and maturation were done in teflon bags; iii) cryopreservation of loaded and matured DC seven days after immunoselection.

Heterogeneity in Tumor Assocciated Antigens (TAA) expression in GB represents the main reason to use whole-tumor lysates as a source of TAA for DC loading. As opposed to synthetic peptides or recombinant proteins, whole cell preparations, such as tumor cell lysate, contain the unaltered spectrum of known as well as unknown tumor antigens that are unique to the patient’s tumor [16].

Our methods to generate tumor lysates are designed to conserve both lipid-soluble and water-soluble molecules as tumor lysis takes place in 0.9% NaCl without detergents. Western blot analysis confirms the presence of both membrane-bound and cytoplasmic proteins (data not shown).

The “classical method” [4], [17] was based on tissues mechanical dissociation with loss of tissue, increased risk of microbiological contamination risk and decreased reproducibility of the procedure. Here, we propose an improved and standardized protein-extraction procedure, which led to an increased protein yield.

As a further confirmation of the improvement provide by the semi-automated method of tumor lysis, we observed a significant increase in CD83 expression on DC activated with the tumor lysate produced following the “new method” rather than the “classical method” tumor lysate.

Moreover flow cytometry analysis performed on DC induced to maturation with cytokine cocktail in the absence of tumor lysate indicates that expression levels of CD83 on mature-non activated-DC (data not shown) are as homogeneus as observed on “new method” lysate activated DC. This result seems to indicate that the non-homogeneous lysates obtained via the old “classical method” reflected in the non-homogeneous expression of CD83. All these drawbacks were overcome by the use of the GentleMACs device.

The state of complete DC activation is known to correlate with high expression of CD83, presently the most specific marker for mature DC. CD83 acts as essential enhancer during T-cell activation and initiation of the primary antitumor immune response [19]. Membrane bound CD83 enhances the *in vitro* generation of cytotoxic T cells and enables the long-term survival of antigen-specific T cell by inducing proliferation and inhibiting apoptosis; moreover the activation of CD83, in turn, promotes MHC-II and CD86 expression on DCs [20].

DC orchestrates a variety of immune responses by stimulating the differentiation of naive CD4 T cells into helper T effectors. Antigen presentation by activated-mature DC enhances T cells responses *in vitro* and *in vivo* [21], [22]. Loading DC concomitantly with TAA and the highly immunogenic protein KLH is reported to enhance the T cell response against tumor cells [16]. KLH also serves as a tracer molecule to monitor the immune response and functions as helper antigen with adjuvant properties. Comparatively, weak tumor reactive T lymphocytes benefit from the stimulatory conditions generated during KLH activation of reactive T lymphocytes [23].

The maturation state of DC is considered a key determinant of the outcome of T cell activation leading to T cell tolerance or T cell immunity [24]. It is important to use maturation protocols which do not lead to terminally differentiated DC, as these cells may transiently produce IL-12, becoming refractory to subsequent in vivo induction of IL-12. PGE_2_ was added to the maturation cocktail in order to avoid ex vivo conditions that induce DC to express IL-12, still assuring their *in vivo* migratory ability [19], [23]. ELISA assay performed on mDC supernatant compared to iDC and aDC demonstrated that our mDC do not release IL-12 (data not shown).

IL-12 DC production is (and will be in the future) a controversial theme. PGE_2_ is essential for the migration (*in vitro* and *in vivo*) of DC to the lymph-nodes. DC stimulated with pro inflammatory mediators (TNF-α, IL-1β, IL-6 and PGE_2_) did not produce (or produce lower) IL-12p70 but demonstrate high expression of CCR7 resulting in strong migration ability [25], [26]. The “standard” maturation cocktail (TNF-α, IL-1β, IL-6 and PGE_2_) is GMP-compliant and can be considered as a good “compromise” between IL-12 production and DC migration.

The functionality of maturation cocktail is confirmed by flow cytometry that, at the end of the procedure, shows high levels of HLA-DR and co-stimulatory molecules on mDC surface [27] revealing that maturation is complete.

DC potency, moreover, was evaluated by their allo-stimulatory capacity measured by MLR test, the “gold standard” to test the functional ability of DC as antigen presenting cells [28], [29]. MLR confirmed that mDC are more potent than their immature counterpart in immune response induction simulated *in vitro* using PBMC as responder cells.

DC were also evaluated *in vitro* for their ability to induce specific T cells responses in the PBMC of GB patients before the start of the vaccination protocol (time zero). None of the analysed patients presented a positive immune response *in vitro* at time zero. This observation is fundamental and preliminary to the immunological follow-up of the patients during and after vaccinations. The evaluation of immunological parameters after mDC vaccination of GB patients is currently in progress.

Previous phase I/II clinical trials on DC immunotherapy for brain tumor have established that this treatment is well tolerated, also using different administration protocols [30] and detected immunological anti-tumor responses [31]. DC derived with our protocol confirm their clinical safety and preliminary data on patients that we have vaccinated show prolonged survival and immune response activation (Pellegatta et al, manuscript under review).

DC-based vaccination in patients with GB is feasible and we believe that tumor vaccines may play an adjuvant role in GB treatment, enhancing their responses to conventional therapy.

## Supporting Information

Figure S1 **DC production process: flow chart.** Schematic representation of the production process, starting from leucapheresis arrival and ending with DCs thawing (batch release). Block on the left are representative for the quality controls performed during, and at the end, of the production process.
(TIF)

Figure S2 **Lysate production flow chart: comparison between old “Classical method” and “New method.”** Schematic representation of the lysate production process (day n. 5). Old “Classical Method” is summarized on the left of the scheme; “New Method” is represented on the right of the scheme. Green (for Classical Method) and Blu (for New Method) blocks highlight the differences between the two methods.
(TIF)

Figure S3 **Controlled-rate freezer curve.** Representative example of controlled-rate freezer curve (Planer Kryo 360-3.3, Planer Products) profile for DC cryopreservation: starting temperature 10°C; 1St ramp: −1.0°C/min until temperature of 1.0°C; 2nd ramp: hold for 5 minutes; 3rd ramp: −1.0°C/min until temperature of −9.0°C; 4th ramp: −20.0°C/min until temperature of −40.0°C; 5th ramp: +15.0°C/min until temperature of −13.0°C; 6th ramp: −1.5°C/min until temperature of −20.0°C; 7th ramp: −1.0°C/min until temperature of −40.0°C; 8th ramp: −5.0°C/min until temperature of −60.0°C; 9th ramp: −10.0°C/min until temperature of −140.0°C; 10th ramp: hold for 5 minutes.
(TIF)
