# Supplementary figures and images for: An Optimized Method for Manufacturing a Clinical Scale Dendritic Cell-Based Vaccine for the Treatment of Glioblastoma
Source: PLoS One. 2012 Dec 20;7(12):e52301. doi: 10.1371/journal.pone.0052301 (PMC3527532; doi:10.1371/journal.pone.0052301)

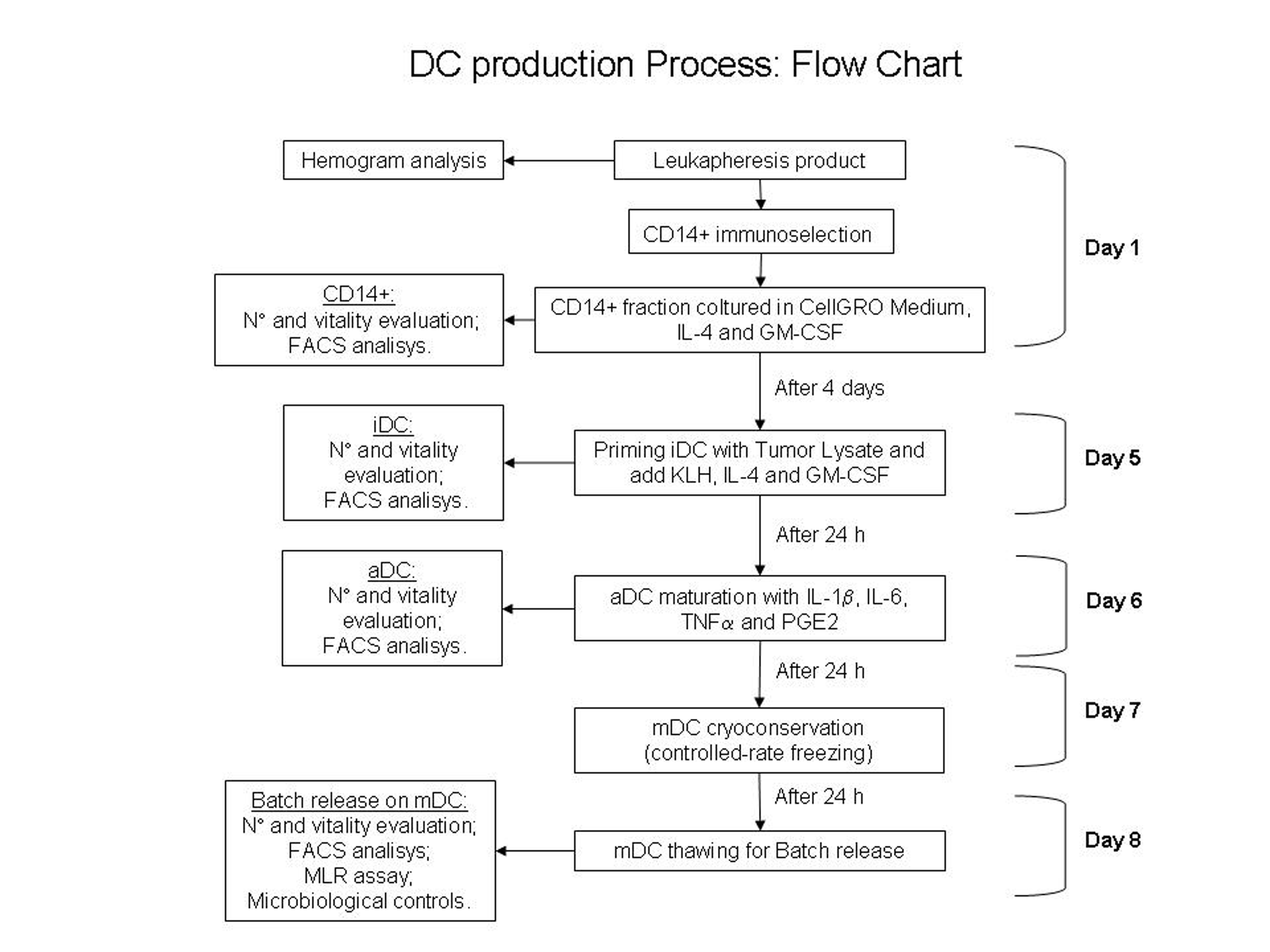

Supplement: Figure S1 — DC production process: flow chart. Schematic representation of the production process, starting from leucapheresis arrival and ending with DCs thawing (batch release). Block on the left are representative for the quality controls performed during, and at the end, of the production process. (TIF) [file pone.0052301.s001.tif]

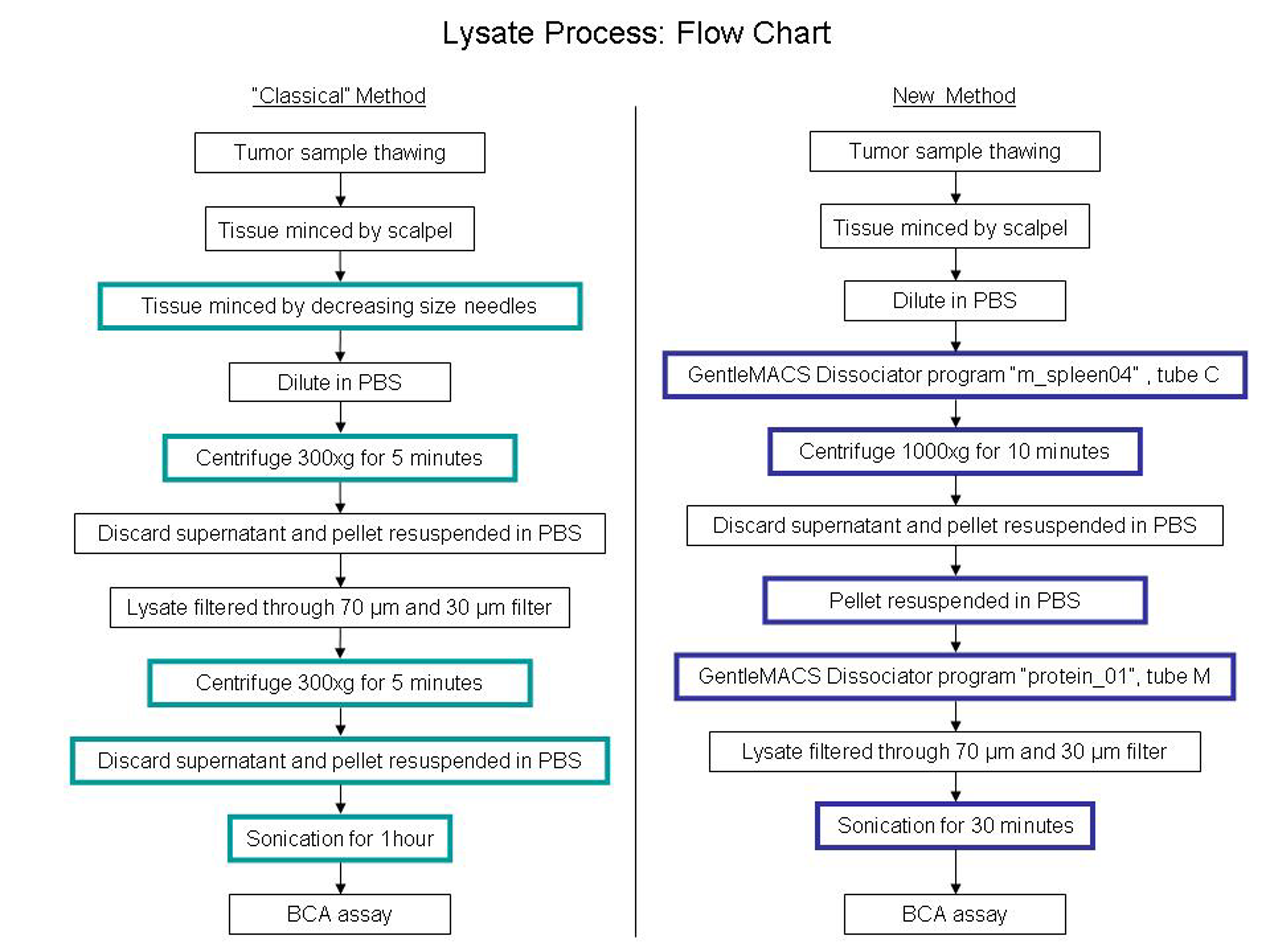

Supplement: Figure S2 — Lysate production flow chart: comparison between old “Classical method” and “New method.” Schematic representation of the lysate production process (day n. 5). Old “Classical Method” is summarized on the left of the scheme; “New Method” is represented on the right of the scheme. Green (for Classical Method) and Blu (for New Method) blocks highlight the differences between the two methods. (TIF) [file pone.0052301.s002.tif]

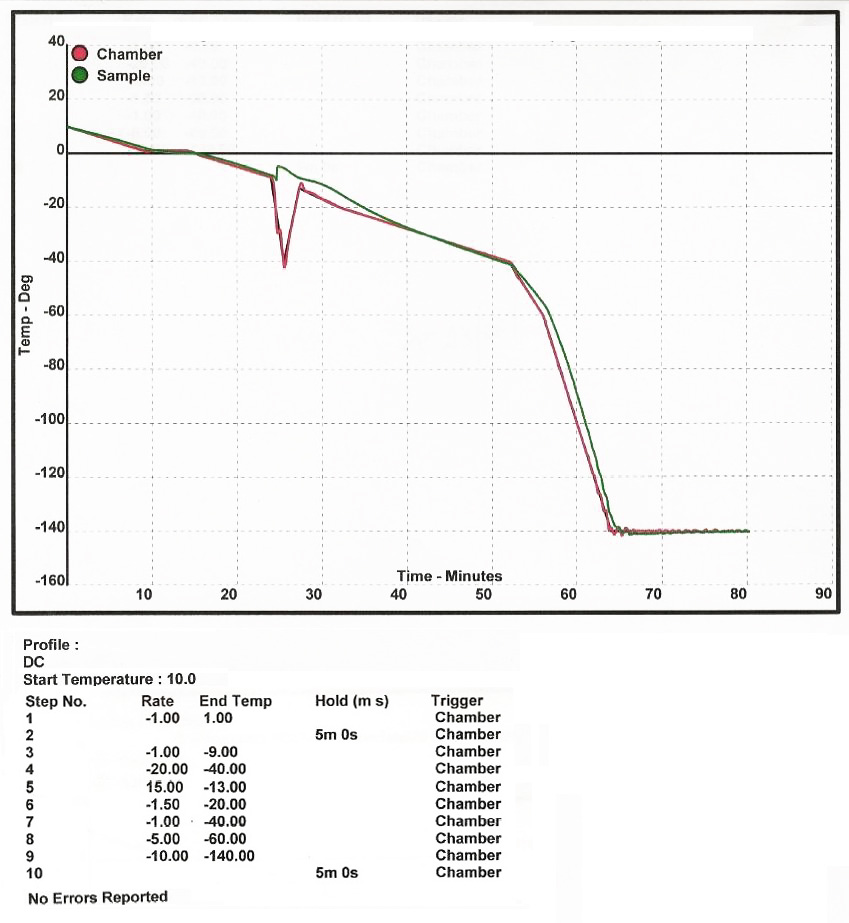

Supplement: Figure S3 — Controlled-rate freezer curve. Representative example of controlled-rate freezer curve (Planer Kryo 360-3.3, Planer Products) profile for DC cryopreservation: starting temperature 10°C; 1St ramp: −1.0°C/min until temperature of 1.0°C; 2nd ramp: hold for 5 minutes; 3rd ramp: −1.0°C/min until temperature of −9.0°C; 4th ramp: −20.0°C/min until temperature of −40.0°C; 5th ramp: +15.0°C/min until temperature of −13.0°C; 6th ramp: −1.5°C/min until temperature of −20.0°C; 7th ramp: −1.0°C/min until temperature of −40.0°C; 8th ramp: −5.0°C/min until temperature of −60.0°C; 9th ramp: −10.0°C/min until temperature of −140.0°C; 10th ramp: hold for 5 minutes. (TIF) [file pone.0052301.s003.tif]
